# Finding shared meaning in the Anthropocene: engaging diverse perspectives on climate change

**DOI:** 10.1007/s11625-021-00965-4

**Published:** 2021-06-05

**Authors:** Gail Hochachka

**Affiliations:** 1 Department of Sociology and Human Geography, Faculty of Social Science, University of Oslo, Postbloks 1096, Blindern, 0317 Oslo, Norway; 2 Present Address: 5613 Montgomery Place, Vancouver, BC V6T 2C8 Canada

**Keywords:** Psychology of climate change, Meaning-making, Constructive-developmental psychology, Photovoice, Ontological pluralism, Transformations to sustainability

## Abstract

The scientific evidence of climate change has never been clearer and more convergent, and calls for transformations to sustainability have never been greater. Yet, perspectives and social opinions about it remain fractured, and collaborative action is faltering. Climate policy seeks to forge a *singular* sense of climate change, dominated by an ‘information deficit model’ that focuses on transferring climate science to the lay public. Critics argue that this leaves out certain perspectives, including the *plurality* of meanings uncovered through participatory approaches. However, questions remain about how these approaches can better account for nuances in the psychological complexity of climate change, without getting stuck in the cul-de-sacs of epistemological relativism and post-truth politics. In this paper, I explore an approach through which we might find shared meaning at the interface of individual and collective views about climate change. I first present a conceptual framework that describes five psychological reasons why climate change challenges individual and collective meaning-making, and also provides a way to understand how meaning is organized within that. I then use this framework to inform the use of photo voice as a transformative (action-research) method, examining its ability to overcome some of the meaning-making challenges specific to climate change. I discuss how participants from a coffee cooperative in Guatemala reflected first on their own climate meanings and then engaged in a meaning-making process with other actors in the coffee value chain. Findings suggest a psychosocial approach to climate engagement—one that engages both subjectively and intersubjectively on the complexities unique to climate change—is helpful in acknowledging an ontological pluralism of ‘climate *changes*’ amongst individuals, while also supporting a nexus-agreement collectively. This may in turn contribute to a more effective and ethical process of transformation.

## Introduction

Global environmental challenges, which are characteristic of the Anthropocene, evade resolution in part because they challenge our meaning-making. Climate change is a prime example. Both the complexity of climate change and its enmeshment with self-identity, culture, values, ideology and beliefs, result in not only a crisis of meaning, but a crisis of *shared* meaning (Hochachka 2019; Hulme 2009; Kahan et al. 2011; Morton 2013; Norgaard 2011; Stanovich et al. 2013; Stoknes 2015). Populations end up very divided about what climate even is, let alone what to do about it (Graham et al. 2014; Maibach et al. 2011; Roser-Renouf et al. 2009). Such varied meanings on climate change can exacerbate existing misunderstandings and contribute to ongoing conflicts between actors with differing worldviews and values (de Witt 2015; Madden and McQuinn 2014). At the same time, the Intergovernmental Panel on Climate Change (IPCC) has put out calls for fundamental, *transformative change* across society to reckon with the climate challenge (IPCC 2018), and ‘climate change engagement’ is increasingly becoming synonymous with engaging with transformation. Yet at the moment when climate science has never been clearer and the calls for transformations to sustainability never louder, the ‘value-action’ gap between what people say and what people do regarding climate change persists and opinions remain fractured (Blake 1999; Climate Action Tracker 2019; Stoknes 2014). In this paper, I propose that better integration of the plurality of individual and collective meaning-making is needed in public engagement strategies, which I argue may in turn support processes of effective and ethical transformations to sustainability.

To date, a common response to the plasticity of climate change meanings has been to assume people simply do not understand climate science correctly. A prominent engagement strategy, therefore, has been to forge a singular, universal understanding of the phenomenon using the ‘information deficit model’ or the ‘empty bucket theory,’ where more and better climate science is transferred to lay publics in a unilateral manner (Stoknes 2015; Suldovsky 2017). This approach has been found ineffective, as it tends to become patched onto prevailing mental frames that either don’t relate with existing ideologies and risk becoming rejected (Feygina et al. 2010), remains cognitively isolated from the inherent knowledges that already exist on the matter (Findlater et al. 2018), or forecloses on the possibility of meanings with alternate ontological or normative underpinnings (Macnaghten 2020). When it comes to climate change, people do not tend to take the findings at face value in the same way they would a more straight-forward issue; rather “‘evidence’ around climate change is searched, remembered, and assimilated in a way that dovetails with people’s own political loyalties and their worldviews” (Hornsey et al. 2016, p. 625). A strategy that people use to understand climate change is to apply heuristics (self-educating techniques), yet these often don’t conform with what a person may cognitively understand about the climate science as much as they seek to placate emotional and cultural knowledge of the situation (Hagerman and Satterfield 2014; Norgaard 2006a). As a result, engagement efforts that insist on proceeding from, and converging others into, a *singular* climate science frame do little to change the underlying worldviews that inform how facts are selected and how the problem is characterized in the first place.

Proceeding from a singular climate frame, which in turn may be used to impose sustainability transformation on publics who have had little say in its design, is also considered unethical (Bennett et al. 2019; Manuel-Navarrete and Pelling 2015; O’Brien and Leichenko 2003). Some scholars call for more participatory, inclusive approaches, moving from individual “multiple cognitions” of personal meaning to interrelated “distributed cognition” of shared meaning (Pahl-Wostl et al. 2007, p. 3). This more relational, reflexive engagement with scientific concepts—i.e. a co-production of knowledge—is argued to be an important manner by which transformation might occur in society (Wynne 2015). Stirling (2014) described how “the most effective modes for radical change often lie in spontaneous collective bottom-up ‘culturings’ of knowing and doing” (p. iii), that “entail[s] more plural, emergent and unruly political re-alignments” (p. 1) and may even contribute to shifting the focus from technocratically-controlled ‘transitions’ to a more bottom-up transformation. Indeed, proponents of such social learning methods suggest these are not just among the deepest hopes for transformation, but also its necessity (Leach et al. 2007; Stirling 2014). However, this paradigm has its own share of persistent puzzles. Scholars warn “against knee-jerk calls for more local, community or public participation which simply replace one set of generalised appeals with another” (Blake 1999, p. 257), as this may risk reproducing some of the very logics that this “pluriverse” tries to side-step (Mercier 2019, p. 9). Pluralizing meanings about climate change may also inadvertently enable an epistemological relativism (made even more fraught in today’s post-truth contexts), where everyone’s subjective truth can be placed on par with everyone else’s, including the scientific ones (Wilber 2017). When this social-learning paradigm attempts to “go beyond the individual level” so as to secure collective outcomes (Vinke-de Kruijf et al. 2014), it may miss important psychological complexities *within* the individual—and unique to climate change—that warrant deeper consideration and integration.

Here, I consider how to make room for a multiplicity of perspectives, not by reducing them into a singular meaning nor by pluralizing all meanings as absolute truths, but rather by asking: “How can a psychosocial approach to individual and collective meaning-making help address different, possibly conflicting, perspectives to realize greater justice and sustainability, specifically when it comes to climate change?” Situated within a larger call for transformations to sustainability, I examine how to integrate five key areas of the psychological scholarship on climate change in a community engagement process using photo voice methodology. I then explore and demonstrate the value of a constructive-developmental perspective in understanding the differences in the ways meaning is organized. Through this empirical example, I propose a possible way to animate existing *means* for transformation in a different *manner*—a manner that honours differences in what climate change means to people within a larger network-understanding in a group. The study site is in the highland coffee region of Guatemala, in which coffee producers live subject to the impacts of climate change and also interact within global value chains with multiple actors from different positions, cultures, and perspectives. This presents somewhat of a microcosm for the larger ‘wicked’ *problematique* that this study addresses. Processes are needed by which people can metacognitively take climate change as an object of awareness, reflect on what it means to them individually, and then identify a more ‘distributed’ cognition as a collective, which in turn supports effective and ethical transformations to sustainability.

## Literature review

### Five ways climate change challenges (social) meaning

The ‘value-action’ gap and social inertia distinctive of the climate challenge, is in part due to a complex interplay of individual and social meaning-making processes (Brulle and Norgaard 2019; Westerhoff et al. 2018). Some scholars posit that this (inter)subjective bottleneck may indeed be equally or more important than the technical one when it comes to climate change action and ought to factor centrally into transformative change processes (Gifford 2011; Grothmann and Patt 2005). Below I review the extensive literature on why climate change is subjectively and intersubjectively challenging, grouped into five categories, summarizing solutions from each category.

1. Climate change is *psychologically distant*, in both space and time; often understood to be happening elsewhere and in the future (Brügger et al. 2015). Unlike the immediacy of weather, which provides context-specific information in the present moment (i.e. sweat on the back, rain on the face), the distant nature of climate change requires people to use mental representations to construe it (Trope and Liberman 2010). Rather than rendering its full complexity, often proxies are used that are psychologically closer and more concrete, such as, snowpack levels, rainfall changes, and losses of local animals and plants (Clifford and Travis 2018). Yet, this matter of distance is complex, and caveats are warranted. For example, as personal values are themselves distant, drawing climate change closer may paradoxically also draw one’s attention *away* from the larger landscape of their values and into some challenging proximate considerations, such as trade-offs, risks, and costs, that are consequences of climate action (Brügger et al. 2015). Threatening information can be overwhelming when it is made proximate and can trigger defensive reactions (Brügger et al. 2015), requiring processes for working with these strong emotions. On balance, bringing climate change closer—for example, through considering the personal relevance and connection in one’s daily life—seems to be called for, as long as attention is paid to these caveats.
2. Climate change also presents higher requirements for *abstract* mental representations (Chu and Yang 2018). However, the capacity to create abstract representations differs depending on people’s meaning-making capacities, and varying degrees of abstraction lead to varying mental models and frames on climate change (Breakwell 2010; Hochachka 2019; Weber 2010). This helps explain confusions between ‘weather’ and ‘climate’—the former is more accessible to people in part because it is less abstract—and some scholars argue that greater understanding of these meaning-making capacities (specifically as studied in developmental psychology) is needed (Hochachka 2019; Lynam 2019). To assist people with abstract concepts, Social Representations Theory (SRT) recommend a two-part process of: (1) *objectification* which entails making what was abstract into a concrete object, “sufficiently dense with meaning,” such that it becomes a natural part of thinking about the issue, and (2) *anchoring* which involves categorizing and linking that new object with pre-existing cognitive frameworks (Breakwell 2010, p. 866).
3. Climate change is *entangled in our affect, self-identity and culture*. For example, Norgaard (2011) finds cultural-identity is set upon certain social values and emotional norms that co-define people’s stable sense of themselves. Threats to that stability by global warming can result in the “social organization of denial” (Norgaard 2006b, p. 374) and even “cultural trauma” (Brulle and Norgaard 2019, p. 1), in which even if people understand the climate change predicament, they may edit their thinking on the issue so “to protect themselves a little bit” (Norgaard 2006b, p. 372). The result of this can be to diminish or deny its implications. Some scholars call for “active open-mindedness,” leaving the cognitive space open for longer to lessen the tendency of collapsing into preexisting opinions (Kahan and Corbin 2016, p. 1). However, these same scholars found that individuals highest in open-mindedness were still polarized on issues like climate change, which seems to have become “tragically entangled in the social dynamics that give rise to pointed, persistent forms of political conflict” (Kahan and Corbin 2016, p. 4). Beliefs about climate change are used by people to express and define themselves and to signal which social group they are a part of, rather than to convey cognitive understanding, and this ought to be carefully accounted for in climate engagement (Kahan 2015).
4. Climate change, and its associated calls for behavioural and social change, is *contested in relation to clashing narratives, values, and interests*, which can lead to complicated trade-offs both intra-psychically as well as interpersonally and politically. Competing narratives about climate change have been advanced, some aimed to protect fossil fuel investments and to deliberately encourage people to hold tighter to beliefs that deny or dismiss the extent of human-caused climate challenge (Moser 2010). This is possible, in part, because people attend to cultural meanings in a parallel manner to the information-content about climate change (Kahan et al. 2011). Moser and Dilling (2011) suggest that democratic citizens would be well served by active engagement on the issue, participating in framing the climate narrative in a culturally congenial manner and rendering more visible the vulnerability of certain groups to climate change.
5. Climate change can get crowded out by other immediate, concrete issues, such that it doesn’t appear on one’s ‘salience landscape’—the mental frame a person cognitively holds to determine relevance and allocate attentional, metabolic, temporal, and behaviour resources (Vervaeke and Ferraro 2013). Inundated by information, people have to expend attentional resources carefully, and climate change can be seen as a *low-salience issue*. This is not new or unique to climate change, and there are known ways to raise the salience of an issue. Much of Freire’s (1970) critical consciousness work sought to facilitate processes by which people could name the world so to transform it—or, rather than living ‘subject to’ a state of oppression, his approach encouraged people to take those dynamics as objects of awareness. Once seen—or made salient—such dynamics could then be acted upon and transformed. In developmental psychology, Kegan (1998, p. 34) explains this process, “mak[es] what was subject into object so that we can ‘have it’ rather than ‘be had’ by it” and he goes on to say, “this is the most powerful way I know to conceptualise the growth of the mind.” This appears similar to how Verveake and Ferraro (2013, p. 39) describe “mindfulness” as being “important for comprehensively transforming and improving the framing of situations so as to avoid becoming trapped in self-defeating construals of situations and problems.” The common thread between these scholars is how to make an issue salient, be that through raising awareness about it, making what was subject into object, or attending to it consciously and mindfully.

### Towards a psychosocial manner of climate engagement

Scholar-practitioners who seek to engage populations on climate change tend to encounter these interlocking meaning-making challenges that are particular to climate change. Often, these challenges are ‘dealt’ with by reducing them into singular climate science (‘one’), which can marginalize important, alternate perspectives, or they are pluralized into multiple meanings (‘many’), which can have an unintended result of undermining science and even paving the way for climate denial. In other words, neither of these approaches are complete, rendering valid an inquiry in climate change communications on how to best support individual and collective meaning-making about such a complex topic.

Finding shared meaning about climate change can be complicated because climate meanings are construed differently by different people, and these constructs have changed over time (Breakwell 2010; Esbjorn-Hargens 2010; Hochachka 2019; Lynam 2012, 2014, 2019; Lynam and Walker 2016). Scholars in the mental models literature emphasize the need to, “unpack the elements that make up the construct of climate change” (Breakwell 2010, p. 859). Constructive-developmental psychology—the study of meaning-making activity (Kegan 1983, 1980)—does so by considering *why* meaning is being organized as it is, beyond the content of *what* is understood about (in this case) climate change. Preliminary research using this approach in climate change suggests that climate meanings are construed differently depending on the complexity of thought that is employed, the object of awareness that is taken (i.e. concrete, abstract, or meta-aware), and the scope of time that is available (present moment, present and past, near future, distant future) (Hochachka 2019). One’s meaning-making apparatus plays a meta-role of coordinating and organizing other data about climate change that are disclosed by the five aspects described above. As such, one’s meaning-making process influences the *distance* at which perception can be wrought out, the *abstraction* of the phenomena in question (from concrete to more subtle to meta-aware), and the extent to which that phenomena “exists” in one’s awareness as *salient* (Hochachka 2019, p. 5). It is also through one’s meaning-making stage that one conceives of their self-identity and how far one’s reach of compassion and care extend, influencing the degree and kind of entanglement in one’s *self-identity and culture* (Graves 1970; Kegan 1980) and one’s *values and worldviews* regarding sustainability (Lynam 2012, 2019). The compound result of these above processes is a mental construction of ‘what climate change means to me.’

Esbjörn-Hargens (2010, p. 148) explains “there is not a clear, single, independently existing object [of ‘climate change’], nor are there multiple different objects [but rather] there is something in-between: *a multiple object*.” Greater recognition of this “ontological pluralism” may open to greater potential for addressing such multifaceted climate change realities in an integrated way (Esbjörn-Hargens 2010, p. 164). “Translating” climate change meanings from existing meaning-making frames may also forge more ownership over such terms, helping to bridge the value-action gap (Hochachka 2019, p. 4). However, while there is extensive research in constructive-developmental psychology in education, leadership, and organizational development (Brown 2011; Cook‐Greuter 2004; Torbert and Barker 2014), it has only minimally been considered in climate change engagement (Hochachka, 2019, 2020). Yet a constructive-developmental lens may help to further explain why people can disagree often vehemently about the issue—namely, *they are seeing different climate changes*. This is a gap I contend with in this paper, in so far as it may help to map collaborative pathways through a plurality of climate meanings.

Seeking to invite *subjective* views as well as support *intersubjective* processes (which I will refer to here as *inter/subjective*), arts-based and participatory approaches, and other transformative action research methods could provide ways to work through these psychosocial challenges particular to climate change. I selected one such method—photovoice—which, when coupled with the following conceptual framework, may bode helpful in enacting the meaning of climate change as “more than one—but less than many” (Mol 2002, p. 55) such that individuals and groups can meaningfully locate themselves in shared climate action.

## Conceptual framework

**Fig. 1:**
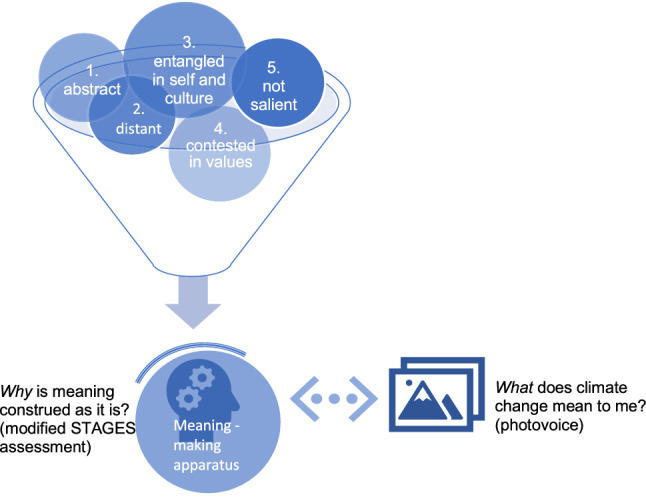
Conceptual framework on how certain aspects challenge people’s meaningmaking processes, leading to a diverse, often contested spectrum of meanings about climate change, which then come together in complex ways in groups

Meaning-making about climate change operates in a rich, layered context of human dimensions, of which at least these five aspects above make climate change psychosocially challenging. Greater acknowledgment of what is affecting individual meaning-making processes at any given time, and thereby indirectly—but importantly—influencing interpersonal processes, may support improved communication and collaboration. I designed Fig. 1 based on the above literature review. The above five dimensions (i.e. distant, abstract, entangled, contested, and not-salient) generate data about climate change, which is then organized by people’s meaning-making apparatus. The latter—namely, how meaning is organized—is less apparent in climate change research and warrants brief description here.

**Fig. 2:**
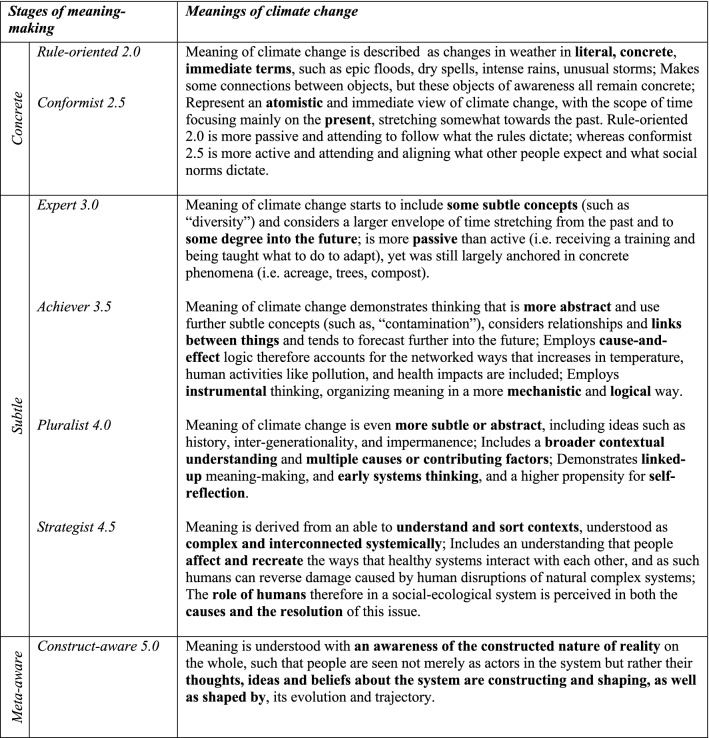
Modified STAGES assessment framework. Describes why meaning is organized as it is assessed by the object of awareness, complexity of thought, and scope of time—based on how much of the complex hyper-object of ‘climate change’ can be seen, at what complexity, via what meaning-making apparatus—drawing on developmental psychology theory as well as empirical findings in a climate change context. Climate meanings are based on Hochachka (2019), whereas stages 4.5 and 5.0 are drawn from applications of developmental psychology in organizational development (Brown 2011; Cook-Greuter 2004; Torbert and Barker 2014)

According to constructive-developmental psychology, meaning is organized in increasingly more complex ways through one’s life, enabled by an increasing ability to take more perspectives on reality (Cook-Greuter 2000; Kegan 1998; Wilber 2000). Preliminary research in a climate change context suggests that more aspects of climate change can become seen with more perspectives taken on it (Hochachka 2019). In Fig. 2, I draw on the STAGES model to describe how these perspective-taking capacities complexify regarding the issue of climate change. The STAGES model is somewhat unique in the broader canon of work on adult development in that it uses assessment logics that focus less on the content of expression and more on the demonstrated perspective-taking capacities that can be seen in the structure of the text (O’Fallon et al. 2020). For example, rather than focusing on *what was said*, much can be understood about the way that a person is organizing meaning that is deeper (or more structural) than the content of the text itself by analysing *how it was said*—namely, demonstrating what subtlety in the object of awareness, degree of complexity of thought, and breadth of time. These perspective-taking stages are titled to approximate the way meaning is construed (i.e. rule-oriented, conformist, expert, achiever, pluralist, and so forth), and, while each have unique characteristics, they are also related to one another in a nested, linked-up way. A developmental perspective honours this spectrum of unique meanings while also recognizing that some contain more complexity than others, as “later stages include perspectives from earlier ones, but not vice versa” (Hochachka 2019, p. 5).

Asking *why and how* meaning is organized as it is, and acknowledging that people construct meaning differently, may provide climate scholar-practitioners with novel entry-points and tools for working with the differences in *what* climate change means to different people. For example, developmental psychology helps to explain one of the fundamental drivers of fragmentation in social groups, namely: *few recognize that their own view of the matter at hand isn’t shared by all*, or that there isn’t a single meaning to which others need to simply get behind. Rather than proceeding from that assumption, developmental psychology instead shows the “*human *being is *meaning making* [and that] for the human, what evolving amounts to is the evolving of systems of meaning” (Kegan 1980, p. 374). Typically people are not cognizant they are organizing meaning—“our meanings are not so much something we have, as something we are” (Kegan 1980, p. 374)—and so for the most part people move through these layered meaning-habitats employing *intuitive* communication skills to connect and understand each other. Yet, this becomes more complicated when working with a hyper-complex concept like climate change. I use this two-part conceptual model (represented in both Figs. 1, 2) to place meaning-making more centrally in a climate engagement process and to examine the inter/subjective factors involved in finding shared meaning about climate change.

## Research design and methods

I sought a research design for this study that could examine the psychological as well as social aspects of meaning-making. I selected photo voice as my main method for its inclusion of subjective and intersubjective processes as well as its ability to reveal the viewpoints of people that may otherwise go unnoticed, thus legitimizing popular knowledge in the face of other dominant discourses (Bennett and Dearden 2013; Hissa 2016; Hochachka 2019; McClymont Peace and Myers 2012; Myers et al. 2012). With photovoice, people use photography to disclose their own subjective perspectives as ‘insiders’ to a region or an issue and to draw those insights into community dialogues, which can then be presented to policy-makers and other actors as a socially- and politically-engaged praxis (Sutton-Brown 2014; Wang and Burris 1997). It has been used after natural disasters to assess local perceptions and to better understand where and how social divisions might arise in rebuilding (Hissa 2016). Most directly relevant to this paper, photo voice was found useful in understanding differences in climate meanings in northern rural El Salvador, by engaging a subjective process of inquiry, an intersubjective process of dialogue, as well as using a modified-STAGES assessment of meaning-construction (Hochachka 2019).

Using photo voice, and its associated methods of interviews and focus groups, I carried out qualitative research with a coffee cooperative, *Association of Agriculturalists “El Esfuerzo”*^1^*of San Pedro Necta* (ASASAPNE), in Huehuetenango, Guatemala, during July 2018 and July 2019, for which ethics approval was granted by the Norwegian Center for Research Data.

Research participants (*n* = 11; 9 women and 2 men) were small producers, meaning they produced coffee in a family-run manner on less than 50 manzanas of land (1 manzana = 7056 square meters, or 1.7 acres). The region is located at approximately 1500 m above sea level, has a largely Indigenous Mam population, and *Arabica* coffee production is a mainstay of the local economy. The cooperative sells a portion of their coffee in a global value chain of a prominent wholesale retailer in North America, with sales also to Taiwan and Italy. The group of participants was diverse in terms of religion (30% Catholic versus 70% Evangelical, which according to Jonas (1991) may have also indicated a difference in past and present political affiliations), gender (82% women and 18% men), culture (36% indigenous Mam, 63% Ladino), age (late-20 s to late-50 s), educational levels (illiterate and minimal education to college-educated), exposure to capacity-building training (i.e. from some being recipients to some being facilitators of such trainings), and differences in cross-cultural and urban–rural experiences (i.e. some being very local and agrarian through to others with extensive cross-cultural, metropolitan experiences including international travel).

Participants took photos in response to two questions about climate change: “What does climate change mean to me?” and “How am I adapting?” I had tested the use of those questions in a previous pilot study and found that they were well-suited to support reflection on climate change in a non-threatening and unique manner. The emphasis ‘to me’ in the first question also carries an epistemological stance of maintaining the “inquirer in every inquiry,” which Montuori (2013, p. 4) described helps to limit possible tendencies toward projection or groupthink, and which French sociologist Edgar Morin (1992, p. 87) reflects is an important “inquiry of oneself on oneself, on reality, and truth.” The photographers spent three days considering the first question and taking photos in response to it. Then, they selected their most significant three photos, downloaded them, and participated in an interview (30 min-1 h) about their photos, providing an interpretation and title for each image (which taken together I refer to as ‘photo-texts’). Then, this occurred again for the second question. The photo voice data consisted of 33 photo-texts for question one and 27 photo-texts for question two. These photo-texts were recorded, transcribed, and translated by native Spanish speakers, and checked by me for accuracy. Transcripts were also checked by the participants.

I then held a series of focus groups, including: (1) a ‘photo forum’ focus group, in which each photographer shared his or her photo-text, and (2) a ‘pattern-finding’ focus group, in which the participants reflected on the entire set of photos, grouped them according to common themes, and engaged in critical dialogue. That was followed by (3) a ‘synthesis’ focus group on these themes and on the process itself held with the photographers, and (4) a ‘sharing’ focus group held in Guatemala city with other actors in the value chain (a very diverse group consisting of a buyer, two exporters, two technical experts, one person from marketing, and the producers from ASASAPNE).

Analysis of the photo voice data began inductively, with a participatory pattern-finding focus group. Such pattern-recognition is well-established in group learning processes (Dozois et al. 2011), and supported reflective, ‘double-loop’ learning on the topic (i.e. examining some of their underlying assumptions) (Argyris and Schon 1978; Mitchell et al. 2012). The analysis then continued deductively using a modified STAGES assessment (Fig. 2) to understand *why* meaning was organized as it was, providing insight into the depth of diversity of these perspectives (Hochachka 2019). 20% of the sample was analyzed by two analysts (myself and Dr. Terri O’Fallon) using the modified STAGES assessment in a blind comparison, resulting in inter-rater validation of within 0.5 of a stage. Finally, I did a qualitative analysis of the focus group data (notes and transcriptions) in NVivo.

### Limitations of the research design

Two limitations in the research design warrant brief discussion. While I had sought a sample that emulated the *complex social terrain* that is distinctive of the climate change discourse, for the photo voice work I selected a sample of research participants from an existing *cooperative* organization. This may have introduced a bias to my findings due to the possibility that the cooperative’s structure predisposed them to work effectively through complex issues, unlike other social groups. However, after reviewing the diversity of this sample (above) as well as considering the benefits of working with a group that was committed, open and interested in the photo voice process, I decided that the pros of using photo voice with a prior-organized group like ASASAPNE, outweighed the cons of them already having an effective cooperative structure. I bore in mind the possibility of this bias in my analysis.

Another limitation was the reliance on linguistic expression for participants to convey meanings about climate change, given the possibility of some language barriers (mainly between Spanish and English; and also, two participants spoke Mam as a first language and then Spanish in a professional setting). I sought to address this limitation in four ways. First, the use of photography helped to bring a non-linguistic lens to the issue of climate change, providing the research participants visual prompts and ways to draw on embodied reflections regarding when, where and why they took their photos. Second, although I have spoken Spanish since 1998, I contracted a Guatemalan research assistant to assist me in understanding any unique phrases or accents. Third, I had a professional translator translate the photo-text interviews, and then reviewed the translations carefully myself. Fourthly, I gave the full transcriptions to the participants for them to check (Birt et al. 2016). However, despite my efforts to mitigate this linguistic limitation, it is reasonable to assume that it could persist in some degree in this study. For this reason, I encourage the reader to understand these results as more of an illustration of the complexifying range of perspectives brought to bear on climate change, viewed in a cross-sectional slice in time, rather than as a fixed, immutable dataset.

## Results

In this section, I share three groups of results from this study: (1) the ten common themes that participants identified in the 60 photo-texts, which show the range of views on *what* climate change means to producers, (2) the six meaning-making stages found in this sample of photo-texts that disclose the depth of diversity in terms of *how* and *why* meaning was construed, and (3) qualitative results from the focus groups on the process itself.

### Finding common themes in a multiplicity of meanings

**Fig. 3:**
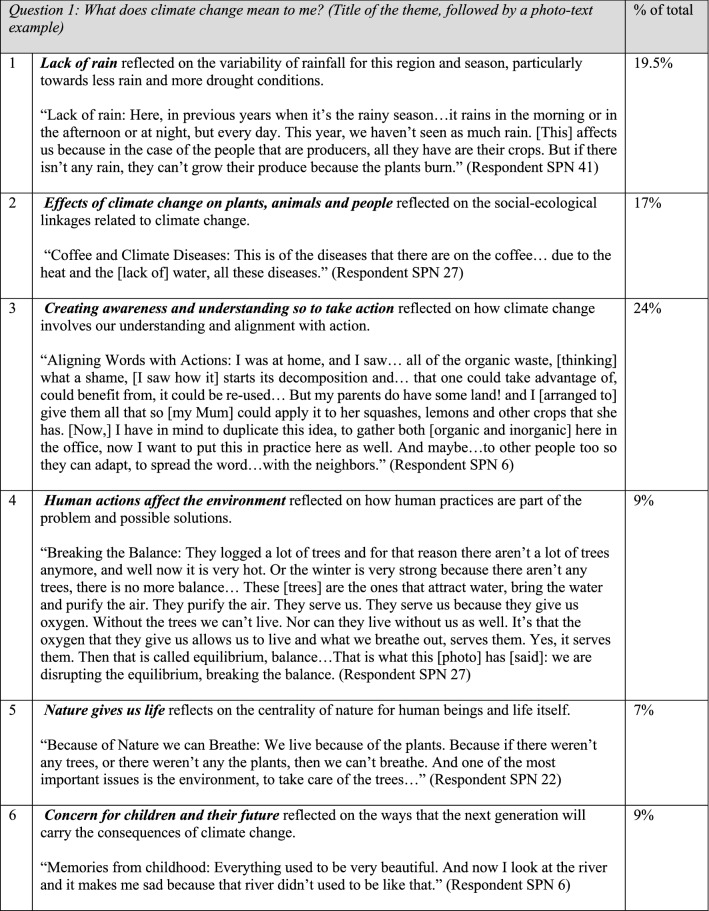

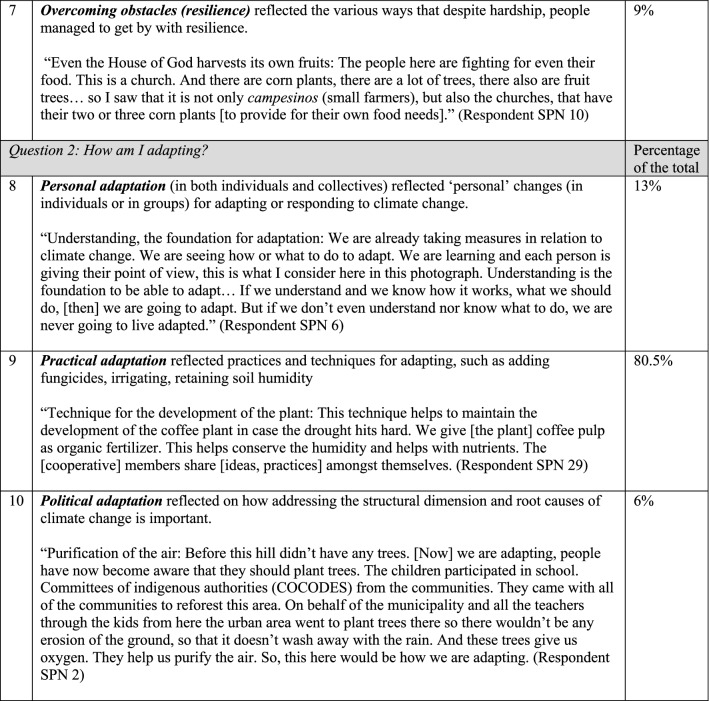
Ten common themes found in 60 photo-texts

The photo voice process resulted in 60 unique viewpoints on the meanings of and adaptations to climate change. Within those, participants identified ten common themes (Fig. 3) (seven themes pertained to photos on the *meaning* of climate change, and three themes pertained to *adaptation*). While many photo-texts were grouped under “Lack of rain,” the largest category was “Creating awareness and understanding so to take action.” Most other themes examined the climate change issue through its social-ecological linkages, examining for example the effects of climate change on both flora/fauna as well as people, the effect humans have on nature, and the ways in which nature give life to humans. Two remaining themes took stock of how resilient people are in the face of hardships born of climate change and considered such hardships for future generations. Themes regarding the question on adaptation were split between three groups, the largest of which was practical adaptation, including how producers are adapting on their farms, with other themes noting the role of understanding (personal adaptation) and advocacy/action (political adaptation).

### Depth of diversity in the constructions of meaning.

**Fig. 4:**
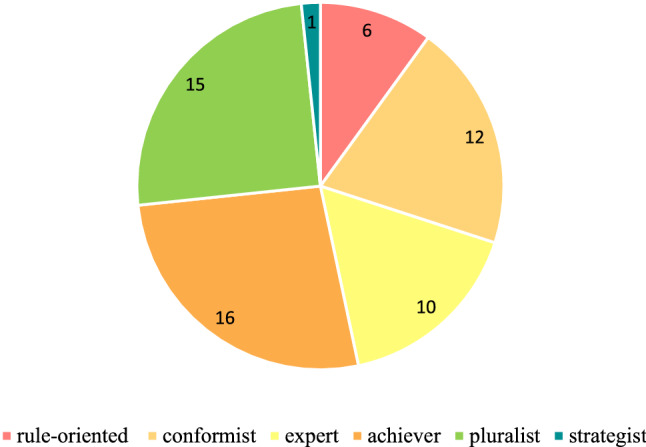
Meaning-making stages represented in the photo voice data for What does climate change mean to me? and How am I adapting? (analyzed with the modified STAGES assessment, *n* = 60)

**Fig. 5:**
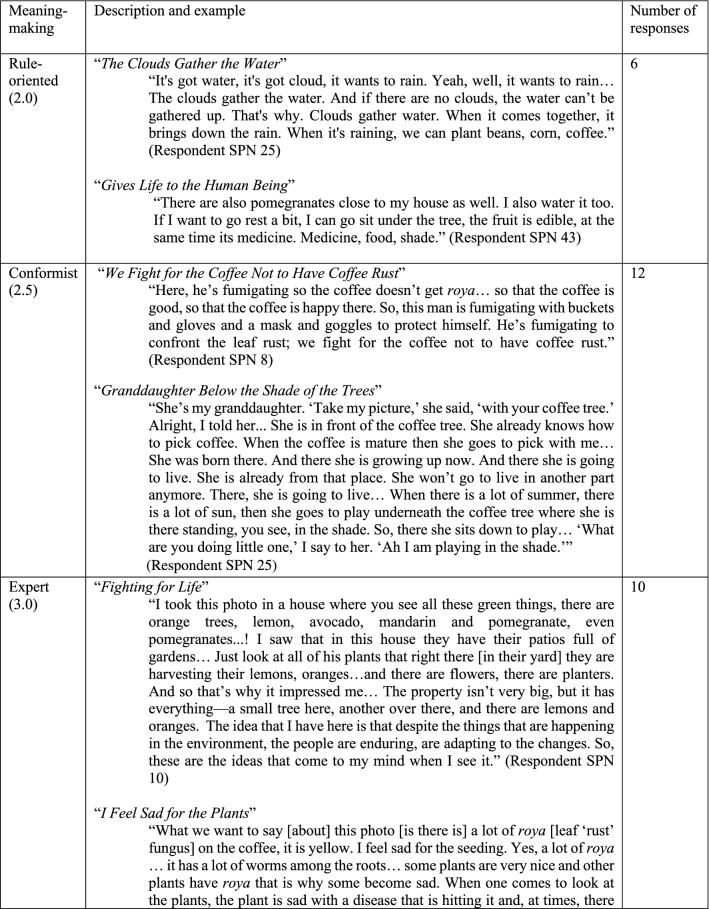

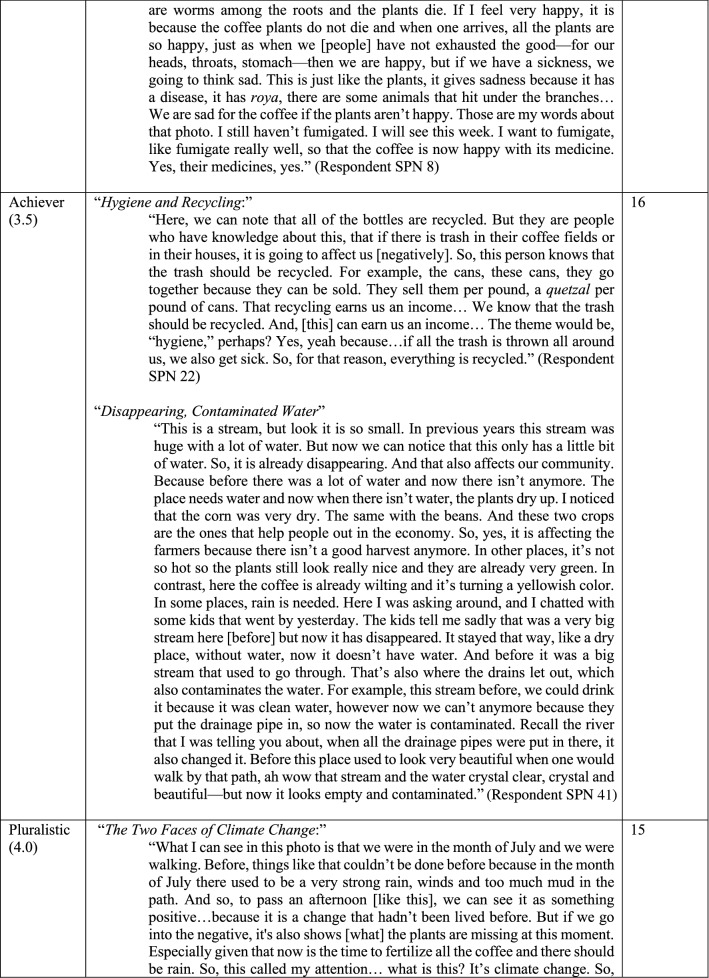

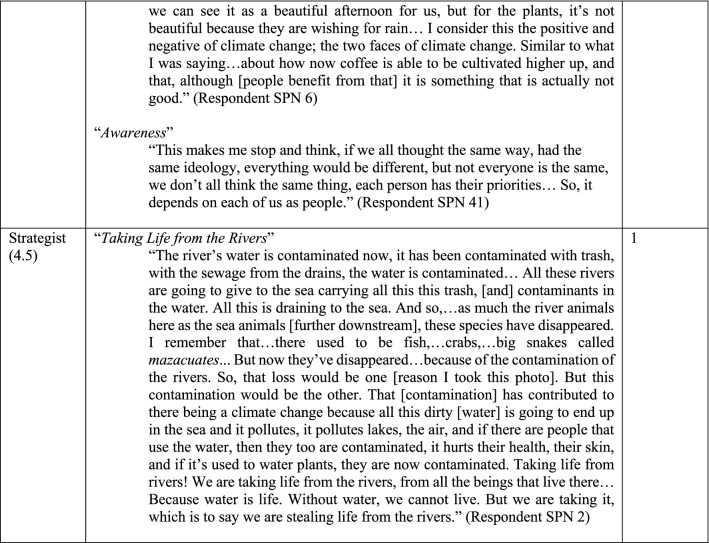
Six stages of meaning-making about climate change. Note these quotes are from self-selected photos taken by participants, and titled and interpreted in their own words. The stages reflect the meaning-making demonstrated in these photo-texts

Six distinct stages of meaning-making were identified (Figs. 4, 5) in the 60 photo-texts that had been taken, titled, and interpreted by the participants. These findings demonstrated the complexification of how meaning is organized about climate change, from more concrete, atomistic organization of meaning through to more subtle, abstract, and networked ways of construing meaning, with the scope of time also differing across the sample. Below, I have presented these six stages in their early and late expressions (2.0 and 2.5 together, 3.0 and 3.5 together, and 4.0 and 4.5 together).

Photo-texts that demonstrated Rule-oriented (2.0) meaning-making construed climate change in a concrete manner, with isolated ‘bits and pieces’ of information that were loosely (if at all) connected to other concepts, largely seen from within the present moment. This meaning-making reflects the static, rule-bound aspects of reality, as this being ‘the way things are’, demonstrated well in the phrase, “And if there are no clouds, the water can’t be gathered up. That's why. Clouds gather water” (*The Clouds Gather the Water*, Fig. 5). Photo-texts that demonstrated Conformist (2.5) meaning-making construed climate change with a concrete reciprocity, within the present moment, often with a traditional, conventionalist framing—such as, “She was born there. And there she is growing up now. And there she is going to live” (*Granddaughter Below the Shade of the Trees*, Fig. 5)—as well as seen in the use of the pronoun ‘we’ rather than ‘I’—for example, “we fight for the coffee not to have coffee rust” (Fig. 5).

Photo-texts demonstrating Expert (3.0) meaning-making showed a concrete cause-and-effect logic with more links made between concepts, using some subtle objects of awareness (i.e. “environment,” “enduring,” and “adapting,” in *Fighting for Life*, Fig. 5). These were construed in the present moment with only a slight stretch into the past and future and demonstrated the participants’ own internalized ideas about something, such as: “The idea that I have here is that despite the things that are happening in the environment, the people are enduring, are adapting to the changes” (*Fighting for Life*, Fig. 5). Photo-texts demonstrating Achiever (3.5) meaning-making projected thinking further into the future, using instrumental, cause-and-effect, abstract logic, and demonstrating awareness of subtle concepts and considered different scenarios in a linked-up manner. For example, in *Disappearing, Contaminated Water* the text considers interlocking aspects of this problem, from quantity of rainfall through to drainage into the rivers, consider subsistence crops, coffee plants, and the economy overall, and considers the state of this system in this moment in comparison with previous years.

Photo-texts demonstrating Pluralist (4.0) meaning-making construed climate change with more context-awareness, such as is seen in the phrase, “not everyone is the same, we don’t all think the same thing, each person has their priorities… So, *it depends* on each of us as people” (*Awareness*, Fig. 5, italics added). With a sense of context, these photo-texts also demonstrated a capacity to see multiple sides of an issue depending on the vantage point; *The Two Faces of Climate Change* from Fig. 5 encapsulate this very well in the phrase, “now coffee is able to be cultivated higher up, and that, although [people benefit from that] it is something that is actually not good.” Photo-texts that demonstrated Strategist (4.5) meaning-making construed the issue as part of a complex adaptive system, organizing meaning in a broader scope of time and space as well as extending one’s sphere of consideration or care for ‘other’ (such as, including river and sea animals, and their ecosystems, humans and other species in *Taking Life from the Rivers*, Fig. 5). Photo-texts demonstrating Pluralist and Strategist meaning-making tended to show greater self-reflection, with the texts including expressions like, “This makes me stop and think” (*Awareness*, Fig. 5).

This data showed that even within a small cooperative, there are still differences in perspectives on climate change, both in terms of what was meant (i.e., the 60 viewpoints reflected in the photos) as well as why meaning was organized as such (i.e., the six meaning-making processes used to construe those meanings). These data also showed that, although participants had had no formal climate education, 42 out of 60 photos (70%) demonstrated either key meaning-making strategies used in climate science (Expert and Achiever) or those that are employed in climate justice and in complex-adaptive systems approaches to climate change (Pluralist and Strategist). Yet approximately a third of the sample were organizing meaning in a way that would not necessarily be resonant with either climate science or climate justice approaches.

### The role of an inter/subjective approach for processing complexity

**Fig. 6:**
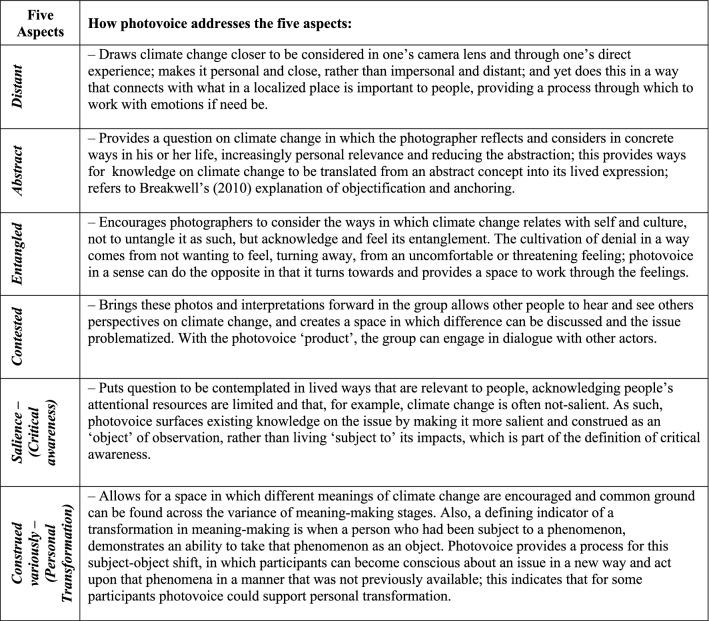
Findings on the usefulness of a psychosocial approach to meaning-making, in this case using photovoice, in learning about climate change in a group

The focus-group transcriptions were analyzed to examine how this psychosocial approach—namely, this two-part conceptual framework combined with the use of an inter/subjective method like photovoice—related with the unique meaning-making challenges of climate change (i.e. distant, abstract, entangled, contested, and not-salient, Fig. 6).

Some quotes noted how this approach enabled them to bring what was *distant*, something only heard about in passing, to consider it in their own direct experience.

“It is a very good technique to be able to analyze and observe how the change has affected the environment and how this change also affects our lifestyle, our crops. We have also learned to contribute and take action to cope with change in different areas of our lives.” (Respondent SPN 2)

This made climate change close and personal, and seemed to do so in a way that kept it connected with what was important to people.

Through contemplating the question ‘what does climate change mean to me?’, participants made climate change less *abstract* by considering the felt-sense and concrete ways that climate change manifests in one’s life:

“It is a technique to have proof to show that climate change is true and to know what is affecting us. To have proof of the changes in rivers, crops, and climate. We can think about living better and having children live well in the future.” (Respondent SPN 27)

Both the photo voice exercise and the dialogue process helped participants visualize the abstract concept of climate change in concrete ways, within their lives.

Other quotes described the ways in which they came to see how climate change was *entangled* with oneself and one’s culture, bound up in one’s own emotions and also linked with broader changes in society across time.

“[Through this process] I learned important things about nature. There are subjects that we avoid but that bring problems. In order not to pollute we must think individually about our actions as they affect ourselves and others. The people of today no longer want to work, they burn the trees, and don't think about the animals and plants that live there. Before the climate was cool but now there is a lot of heat.” (Respondent SPN 41)

This approach provided space to express the emotions that global warming can provoke, such as fear. As one participant said, “There is a lot of clamour about how the climate has changed; we are *afraid* to think about the little ones [children and youth]” (Respondent SPN 25, italics added).

This psychosocial approach also let participants discuss and problematize such ‘avoided’ or *contested* aspects of climate change. Some noted that climate change was also the result of corporations and industrialized countries who produce pollution; others also noted the unequal distribution of vulnerability and risk for the supply-side of the value chain. As explained by one participant:

“I believe we have woken up the observer! Because we are now observers! …now with this, there is something we can do. We know how to change ourselves. To be an example. Some things can be avoided but I believe the contamination is very very broad. And this isn’t something only due to us, the greater polluters are those from industrialized countries, from the large factories, from mining companies. And also the fabrication of all that plastic! We have become habituated to using it, the majority use it because we see it is easier, but on the long term it is actually damaging. But, despite that, they keep fabricating it! They keep making it! So, these other countries should have taken [the responsibility to change]. But at least we can start with ourselves. What we’ve started here, maybe we can become accustomed to it and adopt in other places.” (Respondent SPN 2)

Another respondent echoed this, musing on the use of specifically photo voice in advocacy:

“After this, there will be a history to put into practice in our community or in other regions or countries. We can present the project to the government and other organizations and we can receive more help for the community…to be able to put the study into practice.” (Respondent SPN 12)

One respondent noted the value in this approach for disclosing their own local reality and hearing about others’ realities:

“We will present the research in our own way and others will present [to] us on climate change in their own way. This is an idea to present the reality of our people.” (Respondent SPN 27)

Rather than ignoring the issue, this approach made climate change *salient*, which in turn became important for group learning and action:

“One ignores many things but when seeing the photographs, we realize the reality. It is the truth that the most affected among us is nature and if we do not become conscious about this, we are [all] going to suffer.” (Respondent SPN 42)

“Analyzing the process, is like discovering the problems that exist and becoming aware. Now we know that we must look for solutions since we have the evidence of the problem.” (Respondent SPN 12)

“My view of photo voice is that it is about education and information. When I take the picture, I think about what I can capture and what I can make known in a photo [with] words [that] complement the photograph. The person who listens also learns a lot and captures the meaning of what I want to make known. We find a variety of photography [here] and…by listening to [each] artist’s message you can learn about what he wanted to make known.” (Respondent SPN 6)

Respondents remarked that recognizing this issue in this way then called for action. Respondent SPN 41 said, “now that we have learned about the subject, we have to share what we have learned,” or as Respondent SPN 25 put it, “now, we know and understand about climate change—we are aware and we are going to plant more trees and work more on the coffee—now we understand how we can live better.”

When the actors on the retail side of the value chain bore witness to the perspectives disclosed by producers, in terms of the interlocking stressors of climate change, they came to understand the realities present in the coffee sector in a new way. A buyer who attended the final multi-actor focus-group reflected that the commonality within the variance of views is the central role that humans play as the cause of climate change: “Everyone sees it in a different way, or they see it from a different perspective, but if we take this as a whole, the only one who is responsible [for the fact] that climate change exists is the human being” (Respondent GUA 38). Another respondent, who is a technical expert regarding climate change, was surprised about the producers’ existing climate knowledge and mused on the value of photo voice “as a technique that was not a [formal] technique” (Respondent GUA 36) regarding its capacity to informally and implicitly—but effectively—generate climate awareness and understanding. Indeed, the actors in the value chain had come to know about climate change in a new way through this process, both surfacing existing knowledge—“these are things we knew but didn’t [know we knew]” (Respondent SPN 2)—and extending it across other areas of life—“I have learned during the process that I must take care of the environment, starting with myself and then with my family” (Respondent SPN 22).

## Discussion

This study examined a climate-engagement process that acknowledges the extent to which climate change challenges individual and collective meaning-making, and that might assist in finding shared agreements amidst plural views. The results suggest that the use of photo voice, when carried out in consideration of the psychosocial processes that press upon people as they coordinate their sense-making, is able to honour and include individuals’ meanings as well as convene a network-consensus between multiple actors. This coffee cooperative demonstrated an extensive and ‘deep’ diversity of views about climate change, within which participants convened a shared message that they then brought into generative dialogue with the retail-side of the coffee value chain. In this discussion, I reflect on how this psychosocial approach—one that engages people inter/subjectively, such as was found with photovoice—supported this process of finding shared meaning. I consider first, in Sects. “Bringing climate change closer—reducing distance and abstraction” and “Raising salience by engaging with entangled, contested realities of climate change,” the five aspects that challenge climate meanings; then, in Sect. “From ‘information deficit’ to developing wisdom,” I discuss three types of ‘awareness’ that supported meaning-making; and finally, in Sect. “Creative tensions in collective meaning-making,” I reflect on the coordination of shared understanding within a multiplicity of views on climate change. An approach like this may become increasingly important as climate engagement dovetails with transformations to sustainability and a more effective and ethical manner of community participation is sought.

### Bringing climate change closer—reducing distance and abstraction

Participants considered a typically distant, abstract term like ‘climate change’ and interpreted it through their lived realities through the photo voice process. By phrasing the question in the first-person, participants drew the concept of climate change closer and rendered it at a level of abstraction that was available to them. Mental models research claims that this type of process is important in order to honor peoples’ “intuitive understanding” of climate change within a complex interacting system of beliefs (Breakwell 2010, p. 859). Through what social representation theory calls objectification and anchoring, participants in this study encountered their subjective meanings of climate change as situated within their own cases and contexts. This helped to give ‘density’ to such an abstract concept and helped to bridge the gap between lay and expert knowledge at that individual/collective interface. This collective component is important: “SRT states that objectification and anchoring are not individual processes…[rather they] involve social interaction and the establishment of shared meaning and consensus through communication among people” (Breakwell 2010, p. 866).

In this embrace of multiple cognitions, experts’ scientific knowledge ought not to be displaced, but it does need to map onto existing belief systems, which in turn has been found to support decision-making and action (Breakwell 2010). In this sample, it was notable that, climate science (for example demonstrated in the IPCC materials) is written for meaning-making frames from Expert and later, and the SDGs are considered late-Modern worldview (late Achiever) (de Vries 2019); here, without formal education on climate science as such, over 70% the participants were organizing meaning in a similar way as these large international bodies. Where participants misunderstood aspects of the science of global warming, an inter/subjective method like photo voice could be helpful. For example, in *Contaminated River*, the respondent demonstrated insight in linking plastic pollution with the same fundamental drivers of the climate change issue, yet it appears that there is some confusion on the link between emissions in the atmosphere, plastics, and climate change.

“The river right now almost doesn’t look clean anymore, now everything is contaminated. Before, we used to go down to that river to fish a bit further up. Today, not anymore. I think climate change is coming from the same [place] as the trash, as the plastic, which we thought would protect us, but we know now that *the atmosphere covered the plastic on Earth*. Such that, now here we are [with climate change].” (Respondent SPN 29, italics added)

Considering the meaning-making dynamics at play, this approach helped to first honour the insight present in this statement and then to identify where and how further learning about climate science might be needed.

The risk representation literature suggests “correcting and completing” lay knowledges about a complex issue be carried out in precisely this way: by proceeding from how people mentally construct the issue (Atman et al. 1994, p. 779). For example, in their presentation to the multi-actor focus-group, producers demonstrated the full extent to which they comprehended climate change, not through discrete impacts on coffee production alone, but as a larger suite of impacts on human wellbeing and the natural systems that support life. This eschewed the primary role of climate science to ‘deliver’ this technical understanding, bringing the technical expert to express surprise that the producers had somehow arrived at climate change understanding through the lived inquiry of this photo voice “technique that was not a technique.” This did not mean that the technical expert had nothing to share—on the contrary—but she did so lightly, within the existing latticework of lay-knowledge that had been built through the presentation. This suggests that a psychosocially-informed process like photo voice could provide a synthetic approach, in which climate science meanings become woven within already existing meanings.

### Raising salience by engaging with entangled, contested realities of climate change

This climate-engagement process made visible just how *invisible* climate change can be as one goes through their daily life. As one respondent put it “there are subjects that we avoid,” indicating climate change as one of them. That suggests not that people are unaware of such an issue, but that they avoid their own awareness of it. Due to its size, complexity, and the timelines it operates on, climate change can be pushed to the background by other persistent, simpler, and near-term tasks. This, in part, is due to the fact that attentional resources are finite (Weber and Johnson 2009) while the many demands of life can feel infinite (as the main character in the novel *Flight Behaviour* says, “getting the kids to eat supper, getting teeth brushed…There’s just not room at our house for the end of the world” (Kingsolver 2013, p. 283)). Global warming can get crowded, or selected, out of relevance somewhat as an attention-saving mechanism (Whitman et al. 2018). Shared learning gains in small-scale, highly-deliberate processes may not last once participants return to day-to-day tasks and complicated media landscapes (Findlater et al. 2020).

One of the key successes of this psycho-social approach was its ability to provide a space and process to foreground and observe climate change as an ‘object:’ first, by mooring attention on the central inquiry-questions; then, creating a clearing to examine climate change through photography and dialogue. As climate change moves from what is normally merely ‘part of the water we swim in,’ to a specific object to be considered, different kinds of analysis become available in what is referred to as critical awareness. Participants' comments on the political dimensions of climate change, such as the role of industrialized countries and the larger structural factors at play that make responses to this issue difficult, led to problematizing the issue more broadly. When the producers presented their photo-texts in the final focus, the other actors in the value chain were deeply impacted by the images. It brought up emotions like sadness and a sense of responsibility, seeing the role of humans in global warming and the range of impacts it was causing, affirming that “to name the world, is to transform it” (Freire 1970, p. 88).

### From ‘information deficit’ to developing wisdom

While action research, and photo voice within that, is known to contribute to raising the above Freirean ‘critical awareness’ about the theme in question, results also suggest that this psycho-social process brought forth other *kinds* of awareness as well. For example, one respondent exclaimed, that “these are things we knew, but didn’t [know we knew]” suggesting that a *metacognitive awareness* arose through this process. Metacognition refers to a knowing about knowing, which is a higher-order thinking than bare perception. Researchers have argued that the ‘volatility, uncertainty, complexity and ambiguity’ (VUCA, or ‘wicked problems’) that are characteristic of today’s global issues will require the capacity to “think about thinking” (Fazey 2010, p. 7) or to employ “complex higher-order thinking skills” (O’Fallon et al. 2020). Various innovations in organizational development have precisely ventured in that direction (Conklin 2005; Wilber and Watkins 2015); the field of climate change could do the same, this inter/subjective approach being one possible way.

Secondly, this process engaged people’s sense-making systems in a different way than for example an ‘educational’ training workshop would have (Stedman 2004), something more akin to an “aha” moment that Vervaeke and Ferraro (2013, pp. 28–29) describe as *an experience of insight*. For example, one participant, in contemplating the first question, suddenly recognized that he was holding a ‘larger frame’ on all the questions, one which was guided by the role model of St. Francis of Assisi (the Italian saint who loved nature). This became his first photo—meta to the remaining six photos—that he explained oriented him to the wisdom that he sought to emulate:

“[St. Francis] was the first to call Earth, Mother Earth, and called for us to respect nature… His is a story for us to take on, for us to adopt… He travels with us, like the header of all the other photos; a bigger frame.” (Respondent SPN 27)

It has been said that “by taking the perspective of the sage, one comes to have a salience landscape that is similar to that of that sage” (Ferrari and Weststrate 2012, p. 43). Photo voice—at least carried out in a manner supported by this conceptual framework—provided a scaffolding beyond ‘educating’ on climate change to that of developing wisdom about it.

Thirdly, some participants not only shifted their vantage point but also shifted their perspective from being ‘subject to’ climate change, to reflecting on it objectively. The enduring effect of these subject-object shifts—i.e. dis-embedding from reality and re-establishing awareness from a new perspective—is a central part of the process of *personal transformation* in developmental psychology (Kegan 1998; Wilber 2000). In this study, some participants described how photo voice led them to consider how to embody and apply the new (or newly surfaced) climate understanding, suggestive of an actual personal transformation. Such as, “I have learned during the process that I must take care of the environment, starting with myself” (Respondent SPN 22). The extent of that transformation was not part of this study design but could warrant further investigation.

### Creative tensions in collective meaning-making

The psychosocial application of photo voice in this study provided a space in which people shared their individual constructions of meaning about climate change, and the group overtly acknowledged that range of meanings, pinned across two walls of the meeting room. Within that, participants found the ‘center,’ a set of common themes, which did not serve to erase the other meanings but rather found their overlap.

Seeing all these meaning-systems as essential parts of a whole process of group understanding—which is a central tenant of developmental psychology—changes the quality of the discourse to one of honouring and including, rather than competing and excluding. For example, rule-oriented, conformist, and expert meanings about climate change in this study were crafted in the present moment and considered concrete phenomena with only some links made between concepts; later stages, such as achiever, pluralist, and strategist meanings, were coordinating abstract/subtle concepts in cause-and-effect, context-dependent, and systems-thinking logics, and included the past, present, and distant future. While these later stages included the components of the earlier systems of meaning-making (i.e. concrete objects, present moment), *that was not *vice versa—and yet, *all* these viewpoints contributed unique and important perspectives. This study presents a way in which this can be understood *not as a hierarchy* in which the singular climate-science meaning resides ‘on top’ (and at risk of being unethical and ineffective), *nor as flat* in which all meanings are ‘on par’ (and at risk of epistemological relativism), but rather as a *holarchy*—where earlier whole-systems of meaning become the very parts of later whole-systems of meaning (Koestler 1967; Wilber 1996). Understanding the plasticity of climate meanings as a spectrum of ‘whole-parts’ lessens the charge regarding earlier meaning-systems as being wrong or incorrect, since *they are the parts out of which later wholes are constituted.* The inquiry, therefore, becomes, ‘in what way is this perspective true (even if it is also partial)?’, so to find room for it in the larger whole of group-understanding.

This insight could be helpful for climate change communicators and policymakers working to convene social agreement in multi-actor settings. For example, the broad societal uptake of behavioural- and systems-changes during the COVID-19 pandemic has been more effective than responses to the climate crisis (to date), in part because the pandemic communication strategies captured more of the earlier stages of meaning-making in their messaging (Hochachka 2020). That is, honouring that multiple ‘climate changes’ exist across a linked-up spectrum of views may help to craft a path toward improved collaboration and shared action.

As such, the findings in this study regarding meaning-making suggested that social consensus may be an erroneous target, and rather that what is within reach is *a network-agreement, forged in the center of our overlapping meanings*. This echoes Esbjörn-Hargens (2010, p. 164):

“it seems unlikely that that there will ever be a ‘global consensus’—rather there will be networks of understanding that contain dissenting views and opposite opinions at various scales and within a range of contexts… Climate change is likely just the first of a long string of global issues we will face as a planetary community, so there is an ethical imperative to learn how to address such multifaceted realities in a complex and integrated fashion.”

The final focus group represented the possibility for such a community. With perspectives distributed across many contextual-dimensions—position, gender, income-bracket, cultural background, education level—let alone across a spectrum of meaning-making, the group found each other in the center of those overlapping worlds, bringing care and awareness to discuss what—rendered as ‘more than one, but less than many’—climate change means and what should be done about it.

## Conclusion

Climate change is understood diversely. Using a singular sense of climate change in large-scale transformations to sustainability is neither effective nor ethical, and an alternate, more versatile manner of engagement is needed which can honour the plural views of climate along with that of climate science. This is particularly true at the individual-collective interface, where friction between different views can occur. I brought together certain key areas of the psychosocial climate change literature that explain aspects of why climate change is hard to understand and why it can lead to fractured social opinions, and then used that inter/subjective approach to climate change engagement in a diverse community setting. The study found that by accounting for at least these five psychosocial dynamics as well as the spectrum of ways in which meaning is made, this approach was able to assist participants in holding climate change as both one-and-many, making room for a plurality of perspectives alongside the insights of climate science, while convening a network-agreement for climate action.

## Footnotes

1 The direct translation is “The Effort” but that English translation fails to capture the sense of struggle and liberation that is also part of the term’s meaning, which is why I chose to leave it written in Spanish.
